# Maintenance of Skill Proficiency for Emergency Skills With and Without Adjuncts Despite the Use of Level C Personal Protective Equipment

**DOI:** 10.7759/cureus.7433

**Published:** 2020-03-27

**Authors:** Harsh Sule, Miriam Kulkarni, Gregory Sugalski, Tiffany Murano

**Affiliations:** 1 Emergency Medicine, Rutgers New Jersey Medical School, Newark, USA; 2 Emergency Medicine, St. John's Riverside Hospital, Yonkers, USA; 3 Emergency Medicine, Hackensack University Medical Center, Hackensack, USA

**Keywords:** personal protective equipment, ppe, cbrn, video laryngoscopy, intubation, intravenous, ultrasound, emergency medicine

## Abstract

Objective

To determine the impact of Level C personal protective equipment (PPE) on the time to perform intravenous (IV) cannulation and endotracheal intubation, both with and without the use of adjuncts.

Methods

This prospective, case-control study of emergency medicine resident physicians was designed to assess the time taken by each subject to perform endotracheal intubation using both direct laryngoscopy (DL) and video laryngoscopy (VL), as well as peripheral IV cannulation both with and without ultrasound guidance and with and without PPE.

Results

While median times were higher using VL as compared to DL, there was no significant difference between intubation with either DL or VL in subjects with and without Level C PPE. Similarly, no significant difference in time was found for intravenous cannulation in the PPE and no-PPE groups, both with and without ultrasound guidance.

Conclusions

Existing skill proficiency was maintained despite wearing PPE and there was no advantage with the addition of adjuncts such as video-assisted laryngoscopy and ultrasound-guided intravenous cannulation. A safe and cost-effective strategy might be to conduct basic, just-in-time PPE training to enhance familiarity with donning, doffing, and mobility, and couple this with the use of personnel who have maximal proficiency in the relevant emergency skill, instead of more expensive, continuous, skills-focused PPE training.

## Introduction

The health crises related to Ebola Virus Disease (EVD) in 2014 and, currently, coronavirus Disease 2019 (COVID-19) highlighted a key challenge in caring for patients who have or may potentially have chemical-biological-radiological-nuclear (CBRN) exposures.

Although there are instances where healthcare is deferred until decontamination is complete or the risk of contamination eliminated, there are circumstances where aggressive airway management and hemodynamic stabilization is required with a significant risk of exposure to healthcare providers. Given the high risk of contamination of front-line emergency medicine personnel, the use of appropriate personal protective equipment (PPE) is critical. There are generally two approaches to training exercises - focused training with periodic refresher courses or just-in-time training. The cost burden of preparing for high-risk, low-frequency events such as CBRN incidents is a significant challenge since it places a financial and personnel/time burden on hospitals [1-3]. Moreover, training exercises tend to focus on donning and doffing PPE, and not procedural competence while in PPE. 

In recent years, the use of adjunct devices, such as video laryngoscopy (VL) and ultrasound, has become instrumental in the daily practice of emergency medicine. Conflicting evidence exists in the literature as to whether the use of PPE impedes the ability to simply successfully intubate, and this is further complicated by the impact of VL when using PPE [4-9,10-12]. While there is also conflicting evidence regarding the impact of ultrasound on intravenous (IV) cannulation, there are no studies that address its use with PPE [13-14]. Our study is the first to examine these parameters while using both VL for intubation and ultrasound for intravenous cannulation.

Our primary objective was to determine the impact of Level C PPE on the time to perform intravenous cannulation and endotracheal intubation, both with and without the use of adjuncts. We hypothesized that it would take longer to perform these key procedures while donned in PPE.

## Materials and methods

Ethical consideration

The study was approved by the Institutional Review Board of Rutgers Newark Health Sciences.

Study design

This is a prospective, case-control study with self-matching that was performed in the Extended Treatment Area (ETA) of University Hospital (Newark, NJ), which is part of the emergency department (ED) where all patients with suspected CBRN exposure are evaluated and treated. The subjects were emergency medicine (EM) residents in our four-year residency program that had no previous training related to PPE used but were proficient in the technical skills being evaluated. Each resident served as their own control. All study subjects were consented prior to participation.

Equipment

Participants used PPE certified to provide the maximal level of protection to personnel responding to CBRN agents (Level C). Details of PPE, intravenous cannulation, and endotracheal intubation are shown in Table 1.

**Table 1:** Study equipment NIOSH: National Institute for Occupational Safety and Health; CBRN: chemical, biological, radiological and nuclear

Equipment common name	Manufacturer	Model/Part
Personal Protective Equipment (PPE)		
Powered Air Purifying Respirator (NIOSH CBRN Approved)	3M (St. Paul, MN, USA)	Breathe Easy^TM^ Turbo Unit (022-00-03) Breathe Easy^TM^ Hood (BE – 10BR) Belt (RBE-BLT) Ni-Mh battery (BP-15) Cartridge (RBE-57-CBRN)
Fluid Impervious Coverall w/Hood and Boots	DuPont (Wilmington, DE, USA)	Tychem^TM^ F Coverall (TF169TGY2X000600)
Inner Gloves	Cardinal Health (Dublin, OH, USA)	Esteem^TM^ Stretchy Nitrile (8814NB)
Outer Gloves (Latex)	TIDI Products (Neenah, WI, USA)	TIDIShield^TM^ Powderfree Latex Examination Gloves (BS0460-1)
Outer Gloves (Butyl Rubber)	Showa (Manchester, UK)	Best Butyl Gloves (BST874R3)
Tape	Kappler (Guntersville, AL, USA)	ChemTape^TM^ (99402YW)
Shoe Covers	Onguard Industries (Havre de Grace, MD, USA)	PVC Boot/Shoe Cover (97590)
Intravenous Cannulation		
IV Arm/Mannequin	CAE Healthcare (Montreal, Quebec, Canada)	CAE Blue Phantom^TM^ IV and Arterial Line Vascular Access Ultrasound Model (BPA203-NPN)
Ultrasound	FUJIFILM SonoSite, Inc. (Bothell, WA, USA)	SonoSite M-Turbo^TM^ Ultrasound System L25 Venous Transducer
Endotracheal Intubation		
Airway Mannequin	Laerdal (Stavanger, Norway)	Laerdal^TM^ Airway Management Trainer
Video Laryngoscope	Verathon (Seattle, WA, USA)	GlideScope^TM^ Advanced Video Laryngoscopy (AVL) system

Procedures

Four stations were set up and fully equipped to perform the necessary tasks: two for intubation and two for intravenous access.

**Figure 1:**
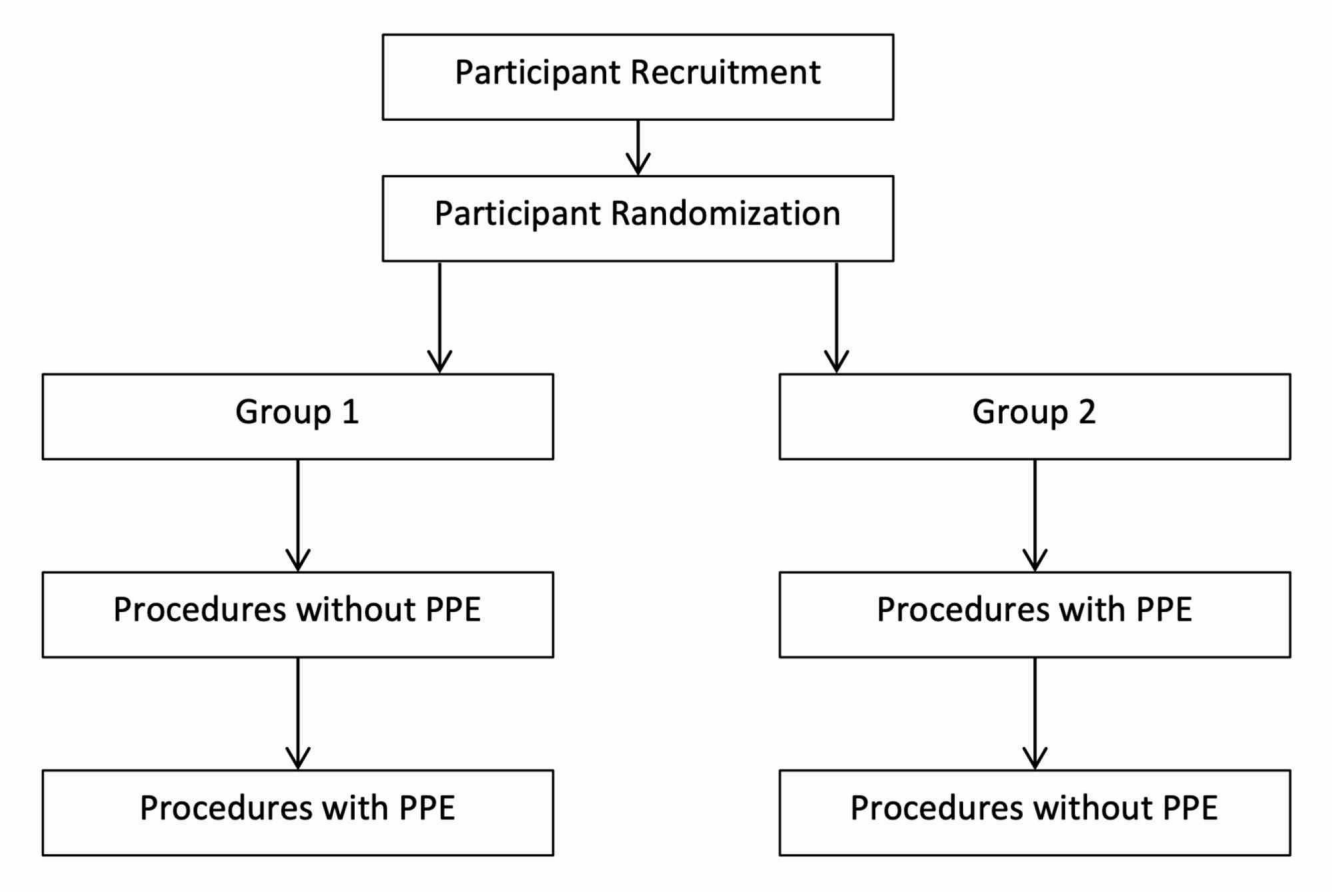
Schematic of study protocol PPE: personal protective equipment

Study subjects were randomized into one of two groups with regard to the sequence of performing procedures, thereby attempting to limit any bias related to the order of procedures. Group 1 performed procedures first without PPE (standard hospital scrubs) and then with PPE, while Group 2 performed procedures first with PPE and then without PPE, as shown in Figure 1.

Half of each EM-year was assigned to each group. Subjects donned and doffed PPE under the direction of experts in the appropriate protocols. Each of the subjects was assigned to one of four procedure stations and rotated in sequence as described in Table 2. Upon conclusion of the study, each subject had attempted each skill twice; once while wearing PPE and once while wearing standard clothing.

**Table 2:** Skill station schedule IV: intravenous cannulation without ultrasound; IV+US: intravenous cannulation with ultrasound; DL: direct laryngoscopy; VL: video laryngoscopy

Group 1								
Subject	No PPE				PPE			
A	IV	IV+US	DL	VL	IV	IV+US	DL	VL
B	IV+US	IV	DL	VL	IV+US	IV	DL	VL
C	IV	IV+US	VL	DL	IV	IV+US	VL	DL
D	IV+US	IV	VL	DL	IV+US	IV	VL	DL
E	DL	VL	IV	IV+US	DL	VL	IV	IV+US
F	DL	VL	IV+US	IV	DL	VL	IV+US	IV
G	VL	DL	IV	IV+US	VL	DL	IV	IV+US
H	VL	DL	IV+US	IV	VL	DL	IV+US	IV
Group 2								
Subject	PPE				No PPE			
A	IV	IV+US	DL	VL	IV	IV+US	DL	VL
B	IV+US	IV	DL	VL	IV+US	IV	DL	VL
C	IV	IV+US	VL	DL	IV	IV+US	VL	DL
D	IV+US	IV	VL	DL	IV+US	IV	VL	DL
E	DL	VL	IV	IV+US	DL	VL	IV	IV+US
F	DL	VL	IV+US	IV	DL	VL	IV+US	IV
G	VL	DL	IV	IV+US	VL	DL	IV	IV+US
H	VL	DL	IV+US	IV	VL	DL	IV+US	IV

Time to successful intubation was recorded for each subject. The procedure start time was recorded when the subject first touched the equipment for preparation. Preparation for intubation included inserting the stylet into the endotracheal tube (ETT), testing ETT balloon inflation, and placing the Macintosh blade onto the laryngoscope handle or the GlideScope^TM ^(Verathon; Seattle, WA) cover onto the light source. The procedure stop time was recorded when the endotracheal tube (ETT) had been correctly inserted in the trachea with initial inflation of the lungs.

Time to successful IV cannulation was recorded for each subject. The procedure start time was recorded when the subject touched the equipment for preparation. Preparation for this procedure included unwrapping the IV catheter from the package, cleaning the surface of the mannequin, placing ultrasound gel, and turning on the ultrasound machine. The procedure end time was recorded upon the successful initiation of a saline flush of the IV line to confirm proper placement.

All procedure times were recorded in seconds (sec) by volunteers who had experience and knowledge of the skills evaluated. Each subject’s times were recorded on standardized data collection forms. No identifying information was recorded on the forms except for EM year. At the conclusion of the study, all forms were collected by the primary investigator. The subjects were then debriefed and given an opportunity to convey their impressions regarding their performance in the skill stations.

Statistical analysis

The Shapiro Wilk test was utilized to determine if the data fit a normal distribution model. Given the small sample size, a two-tailed Mann-Whitney U test was used to compare the time to perform each procedure with and without PPE. Significance was defined as an associated p-value of < 0.05.

## Results

Sixteen of the total 25 eligible EM resident physicians participated in the study. Nine residents were excused because of either scheduling conflicts or work-hour restrictions. Resident participants in the study included two first-year residents (EM-1), 6 second-year residents (EM-2), 3 third-year residents (EM-3) and 5 fourth-year residents (EM-4). One resident’s data was excluded from the video laryngoscopy portion due to incomplete data collection.

Data for all four procedures were found to not fit the normal distribution model. Therefore, median times with interquartile range (IQR) are reported below. When performance time was lower with PPE than without PPE, the time is reported as a negative value.

The median time for successful IV cannulation without ultrasound was 95 sec (IQR 73-117 sec) without PPE and 83 (IQR 31-135 sec) with PPE. The median time difference in performing IV cannulation with and without PPE was -9 sec (IQR -45-27 sec, p = 0.187). The median time for successful intravenous cannulation with ultrasound (IV+US) was 143 sec (IQR 73-124 sec) without PPE and 98 sec (IQR 55-140 sec) with PPE. The median time difference for intravenous cannulation without and with ultrasound was -47 sec (IQR -162-68 sec, p=0.067). The specific values per resident are detailed in Tables 3-4.

**Table 3:** Intravenous cannulation without ultrasound IV: intravenous; PPE: personal protective equipment

EM level	IV without PPE (time in seconds)	IV with PPE (time in seconds)	Change in time to perform procedure with PPE (time in seconds)
1	88	94	6
1	87	114	27
2	109	176	67
2	92	82	-10
2	91	89	-2
2	107	84	-23
2	107	65	-42
2	55	54	-1
3	67	59	-8
3	98	59	-39
3	131	68	-63
4	145	118	-27
4	113	118	5
4	81	53	-28
4	89	115	26
4	206	63	-142
Median	95	83	-9
IQR	22	52	36

**Table 4:** Intravenous cannulation with ultrasound IV: intravenous; PPE: personal protective equipment

EM Level	Ultrasound-guided IV without PPE (time in seconds)	Ultrasound-guided IV with PPE (time in seconds)	Change in time to perform procedure with PPE (time in seconds)
1	121	95	-26
1	129	87	-42
2	151	358	207
2	456	100	-356
2	114	121	7
2	132	433	301
2	184	108	-76
2	112	77	-35
3	62	405	343
3	183	84	-99
3	207	80	-127
4	279	140	-139
4	170	118	-52
4	135	67	-68
4	77	93	16
4	248	71	-177
Median	143	98	-47
IQR	71	43	115

The median time for successful intubation with direct laryngoscopy (DL) was 67 sec (IQR 58-76 sec) without PPE and 54 sec (IQR 30-78 sec) with PPE. The median time difference in intubation via direct laryngoscopy with and without PPE was -9 sec (IQR -29-11 sec, p = 0.159). The median time difference in intubation via video laryngoscopy (VL) was 89 sec (IQR 49-130 sec) without PPE and 86 sec (IQR 51-121 sec) with PPE. The median time difference in intubation via video laryngoscopy with and without PPE was -4 sec (IQR -46-38 sec, p = 0.787). The specific values per resident are specified in Tables 5-6.

**Table 5:** Direct laryngoscopy PPE: personal protective equipment

EM level	Direct laryngoscopy without PPE (time in seconds)	Direct laryngoscopy with PPE	Change in time to perform procedure with PPE (time in seconds)
1	39	68	29
1	74	131	57
2	105	75	-30
2	70	52	-18
2	63	42	-21
2	74	66	-9
2	87	50	-37
2	71	37	-34
3	68	59	-9
3	65	54	-11
3	63	54	-9
4	70	66	-4
4	65	104	39
4	65	34	-31
4	41	41	0
4	49	42	-7
Median	67	54	-9
IQR	9	24	20

**Table 6:** Video-assisted laryngoscopy PPE: personal protective equipment

EM level	Video laryngoscopy without PPE (time in seconds)	Video laryngoscopy with PPE (time in seconds)	Change in time to perform procedure with PPE (time in seconds)
1	74	93	19
1	92	214	122
2	87	119	32
2	128	80	-48
2	59	80	21
2	160	184	24
2	54	32	-22
3	89	79	-10
3	90	150	60
3	54	52	-2
4	255	86	-169
4	98	90	-8
4	80	64	-16
4	70	66	-4
4	291	95	-196
Median	89	86	-4
IQR	41	35	42

**Figure 2:**
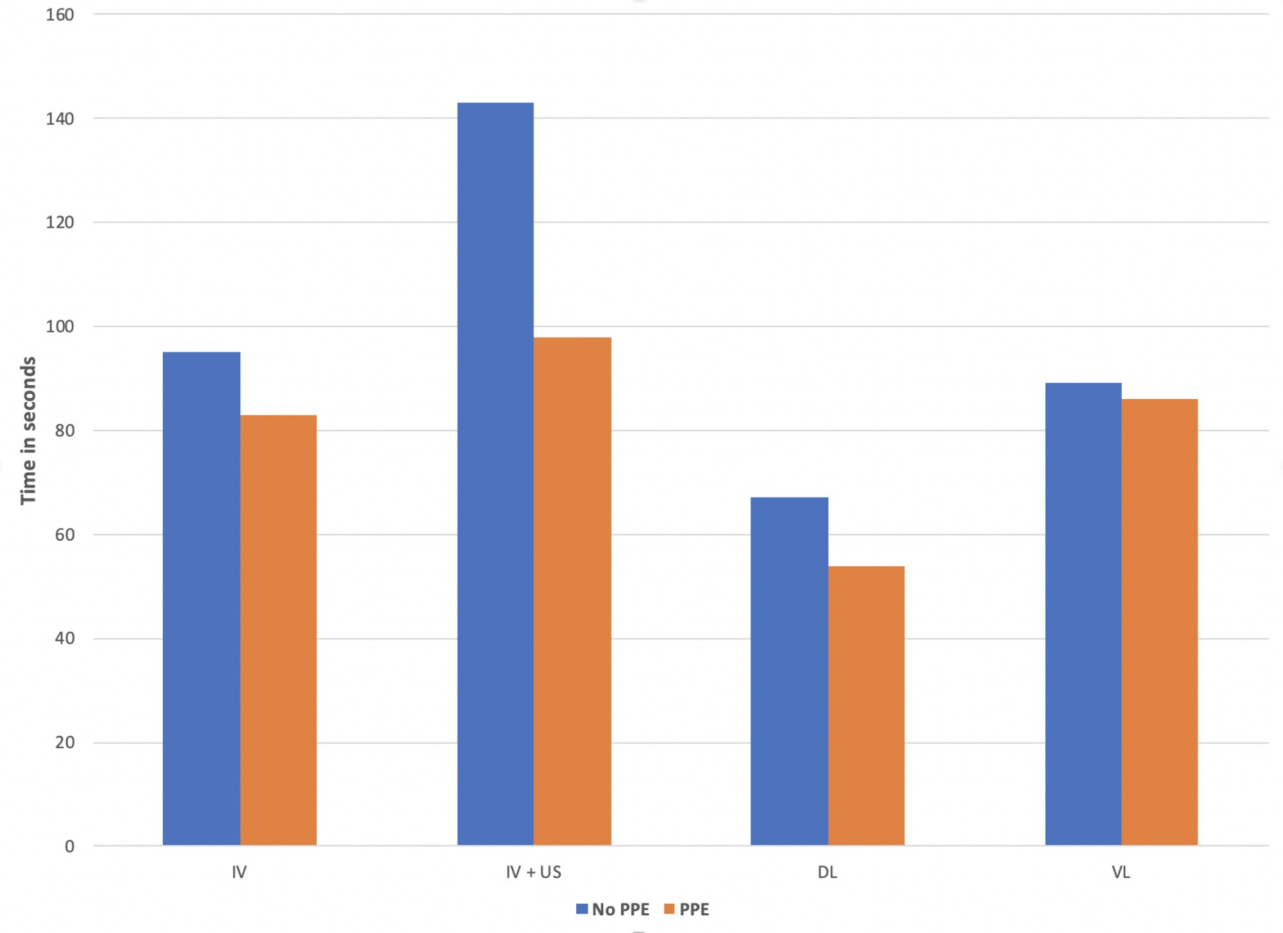
Median difference in time to perform procedure with and without PPE PPE: personal protective equipment

The median time for each procedure with and without PPE is demonstrated in Figure 2.

## Discussion

Our study showed that there was not a significant difference related to Level C PPE use for endotracheal intubation with and without the use of adjuncts. Median times were higher using video laryngoscopy as opposed to direct laryngoscopy, but there was no significant difference in the no-PPE and PPE sub-groups. This is not consistent with several studies where there was an increase in intubation time with the use of PPE.

Flaishon et al. in a study of 15 anesthetists showed a statistically significant increase in endotracheal intubation time when using anti-chemical protective gear (54 ± 24 sec vs. 31 ± 7 sec, P < 0.01) [4]. Garner et al., in a mixed group of paramedics, emergency physicians, and anesthetists noted an increase in time to lung inflation using an endotracheal tube when wearing Level A PPE (78.6 ± 23.9 sec, P = 0.03) [5]. Castle et al. evaluated 64 providers and found an increase in the mean completion time of endotracheal intubation (36.1 sec vs. 67.5 sec) [6].

Consistent with our data, MacDonald et al., in a study of 16 advanced and critical care paramedics, found no statistically significant difference in time to completion of intubation when comparing to a Level C suit (69 sec vs. 79 sec) [7]. In addition, Wang et al. studied 40 emergency physicians (residents) with and without Level C PPE and found no difference in the mean time to successful endotracheal intubation (17.86 sec vs. 17.83 sec, P = 0.99) [8]. Most recently, Adler et al. studied 65 physicians and nurses with varying levels of PPE and found that there were no significant differences in tasks, including endotracheal intubation, except IV placement (median difference, 5.5 sec vs. 42 sec, P<0.01) [9].

We chose to start the time of intubation at the moment the subjects began to prepare equipment for the procedure. Therefore, it is difficult to compare the intubation times in this study with other studies where the start time was post-preparation or insertion of the laryngoscope. However, we felt strongly that this should be included since preparing equipment requires manual dexterity that is influenced by PPE, and in an emergency situation, this preparation will likely be done while donned. Unfortunately, there is limited and somewhat conflicting literature that addresses the question regarding the appropriate time needed to successfully complete airway tasks by otherwise procedurally competent personnel while wearing PPE [4-8]. In our study, the median times for successful intubation with DL and VL (including preparation for intubation), regardless of the use of PPE, were 67 seconds and 89 seconds, respectively. We feel that a time under one and half minutes for preparation and successful endotracheal intubation is an acceptable timeframe.

Similarly, our study showed no significant difference in time for IV cannulation in the no-PPE and the PPE groups, both with and without ultrasound guidance. Although it was not a statistically significant finding, it was interesting that the median times for IV cannulation were faster with PPE than without PPE. Castle et al. found an increase in the mean completion time of IV cannulation when wearing PPE Level C (40.8 sec vs. 129.6 sec) [6]. MacDonald et al. found a statistically significant increase in completion time for IV cannulation when wearing PPE (158 sec vs. 220 sec, P < 0.01) [7]. There has also been no previously established appropriate time for IV placement using PPE; however, the median time for IV cannulation with ultrasound using PPE was 98 seconds. We feel that successful IV cannulation under two minutes is an appropriate time frame.

However, our study has a few limitations. First, the participant group was small thereby making statistical analysis challenging. As a result, we were unable to parse out subtle differences in proficiency that might occur across varying training levels. Second, we did not track the time taken for each individual stage of the procedure; that is, specific time for preparation, time from the insertion of the laryngoscope to passing the ETT and lung inflation. This would have been beneficial in making a direct comparison of our results to existing literature. Finally, while our participant group of trainees completed the procedures in what we consider an appropriate time frame, future studies should include a group of experienced clinicians so that a “gold standard” can be introduced for comparison.

## Conclusions

In this study, we demonstrate that there is no significant difference in completion time for any of the studied procedures with and without Level C PPE, with no advantage related to the use of adjuncts such as ultrasound and video laryngoscopy. Maintenance of existing skill proficiency while wearing PPE is a key finding and perhaps obviates the need for continuous, skills-focused PPE training. A safe and cost-effective strategy might be to conduct basic, just-in-time PPE training for personnel who have maximal proficiency in the relevant emergency skill.
